# Neurologic deficit due to vertebral body osteophytes after oblique lumbar interbody fusion: A case report

**DOI:** 10.1097/MD.0000000000028095

**Published:** 2021-12-17

**Authors:** Tae-Kyu Lee, Jae-Young Kim, Moon-Soo Han, Jung-Kil Lee, Bong Ju Moon

**Affiliations:** Department of Neurosurgery, Chonnam National University Hospital and Medical School, Gwangju, Republic of Korea.

**Keywords:** foramen, neurologic deficit, OLIF, osteophyte, risk factor

## Abstract

**Rationale:**

In recent years, oblique lumbar interbody fusion (OLIF), which uses a window between the peritoneum and the iliopsoas muscle to split the muscle to access the lumbar spine, is known as an effective and safe treatment for spinal diseases, such as degenerative disc disease, spondylolisthesis, recurrent disc herniation, and spinal deformity. Despite this fast and useful surgical method, there were often cases of new neurological symptoms or worsening of symptoms after surgery. We analyzed the preoperative risk factors in a patient with neurologic symptoms, such as motor weakness and exacerbation of radiating pain, after OLIF.

**Patient concerns:**

A 78-year-old man presented with complaints of numbness in the soles of both feet. L4–5 stenosis was diagnosed on MRI. We performed bilateral L4 laminotomy and L4–5 percutaneous posterior screw fixation after L4–5 OLIF. Postoperatively, his radiating pain improved, and there were no other neurologic symptoms. In the 6th week after surgery, he complained of pain in both ankles, while in the 10th week, the pain progressively worsened, and there was a decrease in motor performance of the right ankle.

**Diagnosis:**

Magnetic resonance imaging findings indicated that L4–5 stenosis was resolved. On the basis of the computed tomography findings, the cage was well inserted, the disc height and foramen height increased, and the alignment was good. However, a nerve root injury due to the protruding osteophyte from the inferior endplate of the L4 body was suspected, necessitating exploration of both L4 nerve roots by focusing on the right side.

**Interventions:**

We performed right facetectomy and right foraminotomy. During surgery, it was confirmed that the right L4 nerve root was entrapped by the osteophyte.

**Outcomes:**

Postoperatively, his radiating pain improved, and motor performance of his right ankle was restored.

**Lessons:**

A prominently protruding osteophyte is assessed as a possible risk factor for the development of new neurologic deficits after OLIF. In patients with confirmed osteophytes, surgery should be planned taking into consideration the shape of the osteophytes and their relationship to the nerve root.

## 1 Introduction

Oblique lumbar interbody fusion (OLIF) is an effective and safe surgical method that is widely used for treating degenerative disc diseases to correct various deformities. It is very effective in correcting sagittal and coronal deformities, especially in cases of lumbar degenerative scoliosis with latero-listhesis. OLIF does not require posterior surgery, such as laminectomy or facetectomy, or destruction of the posterior column of the spine and posterior tension band.^[1]^ Recent studies suggest that OLIF is a low-morbidity, reliable, and effective method for the treatment of degenerative lumbar stenosis and spondylolisthesis, as it allows rapid recovery and ambulation after surgery.^[1,2]^

However, despite its many advantages, OLIF is controversial owing to the related inability to decompress the nerve root directly and other complications, such as muscle and vascular injuries, lumbar plexus injury, and urethral injury.^[3]^ The incidence of motor nerve injury after OLIF is approximately 1%.^[4]^ Several studies have reported that lumbar plexus injury or nerve root elongation causes motor weakness.^[5,6]^ Although there is no evidence of injury of the lumbar plexus or nerve roots during OLIF, it is known that motor weakness occurs in some cases after OLIF, and there are currently no studies with an exact analysis. Therefore, we aimed to analyze the risk factors of motor weakness after OLIF in a patient with neurologic symptoms.

## 2 Case presentation

**Figure 1:**
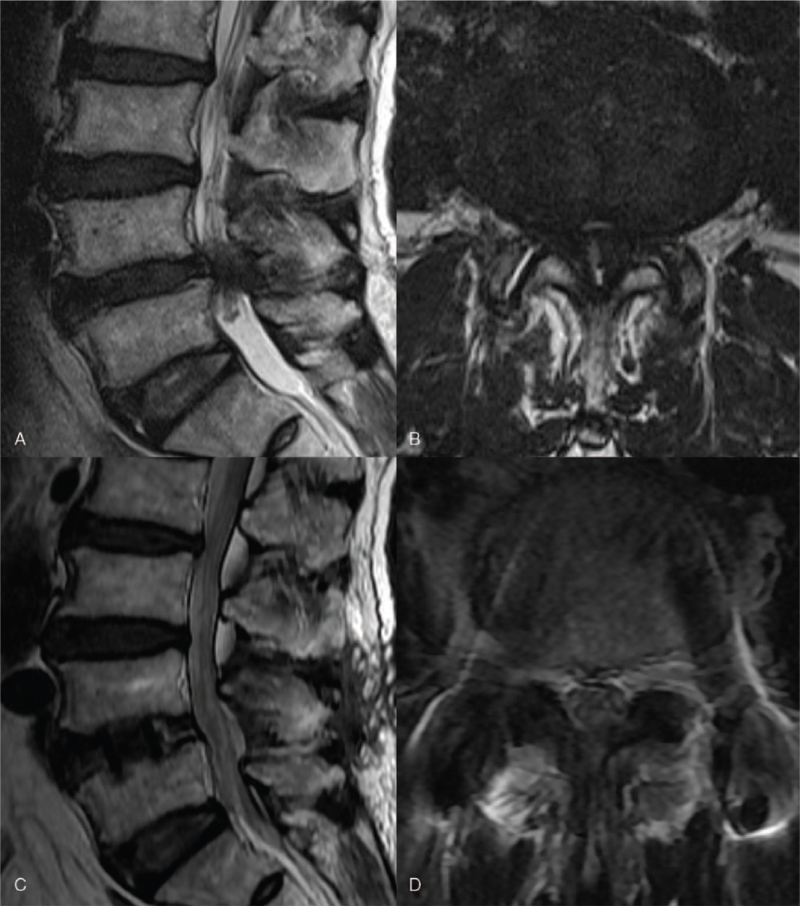
Comparison of the MR images before and after the first surgery (oblique lumbar interbody fusion, followed by bilateral L4 laminotomy and L4-5 percutaneous posterior screw fixation). In January 2018, the patient complained of numbness and pain in the soles of both feet. There was no muscle weakness. (A) Nerve root redundancy is observed in the sagittal view, and (B) severe L4-5 central stenosis is observed in the axial view. In May 2018, the patient presented to the hospital with persistent pain in both ankles and decreased muscle strength in the right ankle. (C) Nerve root redundancy is resolved (sagittal view). (D) Central stenosis is also resolved (axial view).

In January 2018, a 78-year-old man presented with complaints of numbness in the soles of both feet. There was no motor weakness on neurologic examination. Severe L4-5 central stenosis and root redundancy were diagnosed on lumbar MRI (Fig. 1).

On February 28, 2018, we performed L4-5 OLIF, followed by bilateral L4 laminotomy and L4-5 percutaneous posterior screw fixation (PPSF). To perform OLIF first, after administering general anesthesia, we ensured that the hip joint was not flexed in the standard right posture. The axilla and hip were fixed using a wide cloth and tape. The external oblique, internal oblique, and transverse abdominal muscles were incised and approached through the retroperitoneum to gently retract the abdominal organs and psoas to expose the intervertebral space. There was no direct blood vessel injury or nerve injury during this process. After determining the height and length of the cage using the C-arm, we vertically inserted a cage (Clydesdale DLIF Cage, 6°∗14∗50 mm; Sofamor, Memphis) combined with a demineralized bone matrix into the intervertebral space. After turning the patient to the prone position, bilateral laminotomy was performed, with minimal damage to the posterior tension band, and severe ligamentum flavum hypertrophy was confirmed and removed. Thereafter, L4-5 PPSF was performed.

Intraoperatively, an increase in the disc height and foramen height was confirmed using the C-arm, and sagittal alignment was maintained well. There was no motor weakness postoperatively. Two days later, the patient was allowed to walk while wearing an orthosis, and the radiating pain improved; he was then discharged from the hospital.

**Figure 2:**
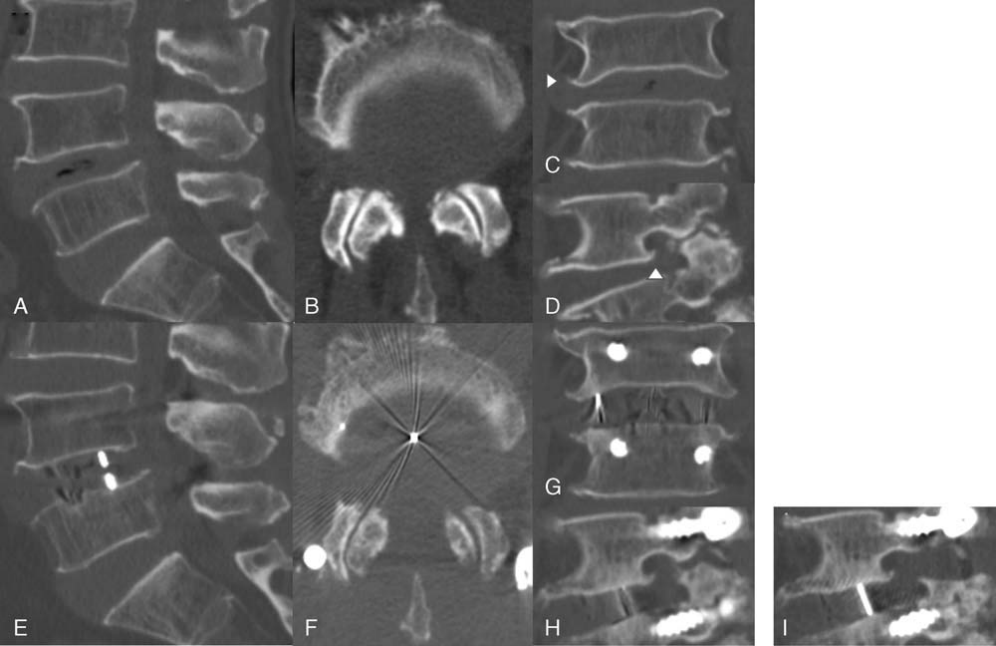
Lumbar CT images before oblique lumbar interbody fusion in February 2018 (A–D) and 10 weeks after the first surgery in April 2018 (E–H) and after the second surgery (I). We measured and compared the anterior, middle, and posterior disc heights in the sagittal plane. We also compared the foraminal height by measuring the vertical distance from the top of the foramen to the superior end plate of the L5 body. The white arrowheads point to the osteophytes we focused on. A: sagittal plane, B: axial plane, C: coronal plane, D: right foraminal view. E: sagittal plane, F: axial plane, G: coronal plane, H: right foraminal view. The height of the anterior, middle, and posterior discs increased by 10% (16.17– 17.73 mm), 32% (13.87–18.26 mm), and 6% (10.95–11.56 mm), respectively, while the height of the right foramen increased by13% (19.36–21.93 mm). Figure 2I presents the right foraminal view after right facetectomy and right foraminotomy, it can be confirmed that the lamina and calcification tissues around the foramen were clearly removed.

At 6 weeks after surgery, he complained of pain and swelling below both ankles at the outpatient clinic. Lumbar CT and radiographic examination showed that the fusion was maintained without subsidence of the surgical site, the height of the intervertebral disc space and foramen increased, and there was no abnormality in the alignment between the lumbar vertebrae (Fig. 2). In addition, a prominently protruding osteophyte, which was considered insignificant before the first surgery, was again observed. In particular, the osteophytes on the foraminal side were severely protruding (Fig. 2). In the 10th week after surgery, he visited the outpatient clinic with a cane, complaining of persistent pain below both ankles and motor weakness of the right ankle. A marked decrease in dorsiflexion of the right ankle was observed. The Medical Research Council (MRC) muscle strength grade of the right ankle was 2. We confirmed the resolution of the previous central stenosis and root redundancy on lumbar MRI and found no other specific findings (Fig. 1).

We thought that it was necessary to explore the L4 nerve roots again, focusing on the right side. On May 21, 2018, we performed right L4/5 facetectomy and foraminotomy to release the right L4 nerve root. During surgery, the tissues compressing the L4 nerve root and the osteophyte protruding severely and trapping the nerve root were identified and removed. Within 1 week of hospitalization after surgery, the radiating pain in both feet disappeared, and dorsiflexion of the right ankle was restored to MRC grade 4. After the second surgery, follow-up CT of the lumbar spine confirmed clear decompression of the right foramen (Fig. 2). To date, he has no radiating pain in both feet, and motor performance of the right ankle has been restored and maintained.

## 3 Discussion

Minimally invasive lumbar interbody fusion techniques are advancing rapidly. Among them, OLIF takes advantage of the lateral approach to access the lumbar spine, enabling a minimally invasive approach for interbody fusion with lower rate of associated morbidity.^[7]^ Compared with other direct decompression surgeries, such as posterior lumbar interbody fusion, OLIF reduces bleeding by minimizing incisions, does not damage the paraspinal muscles and spinal column, and does not directly affect the nerve roots, thus improving postoperative pain and enabling more rapid recovery.^[1,8]^

Despite these significant advantages, there are inherent complications associated with the oblique approach itself, including vascular injury, temporary or permanent injury to the lumbosacral plexus, sympathetic chain injury, vertebral endplate fractures, postoperative thigh sensorimotor impairment, and psoas or quadriceps muscles weakness.^[3,9,10]^

Among these complications, symptoms of neurologic injury include motor weakness, transient sensory change, and sympathetic chain injury, with an incidence of approximately 4.68%.^[11]^ In the study by Zeng et al,^[11]^ the postoperative symptoms observed were pain and numbness in the thigh and transient weakness of the psoas and quadriceps muscles, all of which recovered within a week. Fujibayashi et al^[4]^ reported that the incidence of motor nerve injury after OLIF was 1%, while the incidence of sensory nerve injury was 3.5%. Within 3 months, 65.6% of motor nerve injuries and 69.1% of sensory nerve injuries recovered spontaneously.

Several studies have also reported that neurologic deficits are caused by lumbar plexus injury.^[12]^ Abel et al^[13]^ reported 2 types of mechanisms of lumbar plexus injury. One is direct injury, in which direct laceration or blunt nerve trauma can occur during initial psoas muscle dissection and dilation, discectomy, or insertion of hardware. The other is indirect injury, in which nerve injury is thought to occur by focal compression and/or traction to the nerve during psoas muscle dilatation, retractor placement, and prolonged retraction.

In these previous studies, it is important to note that postoperative complications persisted after surgery. In our study, there was no appreciable direct nerve damage during OLIF and no neurologic deficit immediately after surgery. At discharge, the patient's VAS score decreased from 7 to 2, and the lower back pain and radiating pain improved, which was maintained for 6 weeks.

On the basis of the patient's progress, we judged that it cannot be regarded as a direct injury related to the surgery. Instead of nerve injury as suggested in previous studies, a different hypothesis was needed for the cause of pain and motor weakness that progressed 1 month after surgery.

Other causes were suggested for motor weakness in one study. Dowlati et al^[14]^ suggested that an excessive increase in the height of the disc space causes stretching of the nerve roots, resulting in neuropraxia. In their study of patients with nerve root injury, the anterior and posterior disc heights increased by 134% and 92%, respectively, while the foraminal height increased by 50%. Controversially, in our study, the anterior, middle, and posterior disc heights increased by 10%, 32%, and 6%, respectively, while the foraminal height increased by 13% (Fig. 2). Previous studies have suggested improvements in pain and functional outcomes after fusion, with up to 70% increase in disc height and 33% increase in orifice height.^[15–19]^ On the basis of these findings, it cannot be concluded that the neurologic deficits in our patient were caused by lumbar plexus injury or nerve root stretching due to excessive increases in the disc height and foraminal height.

**Figure 3:**
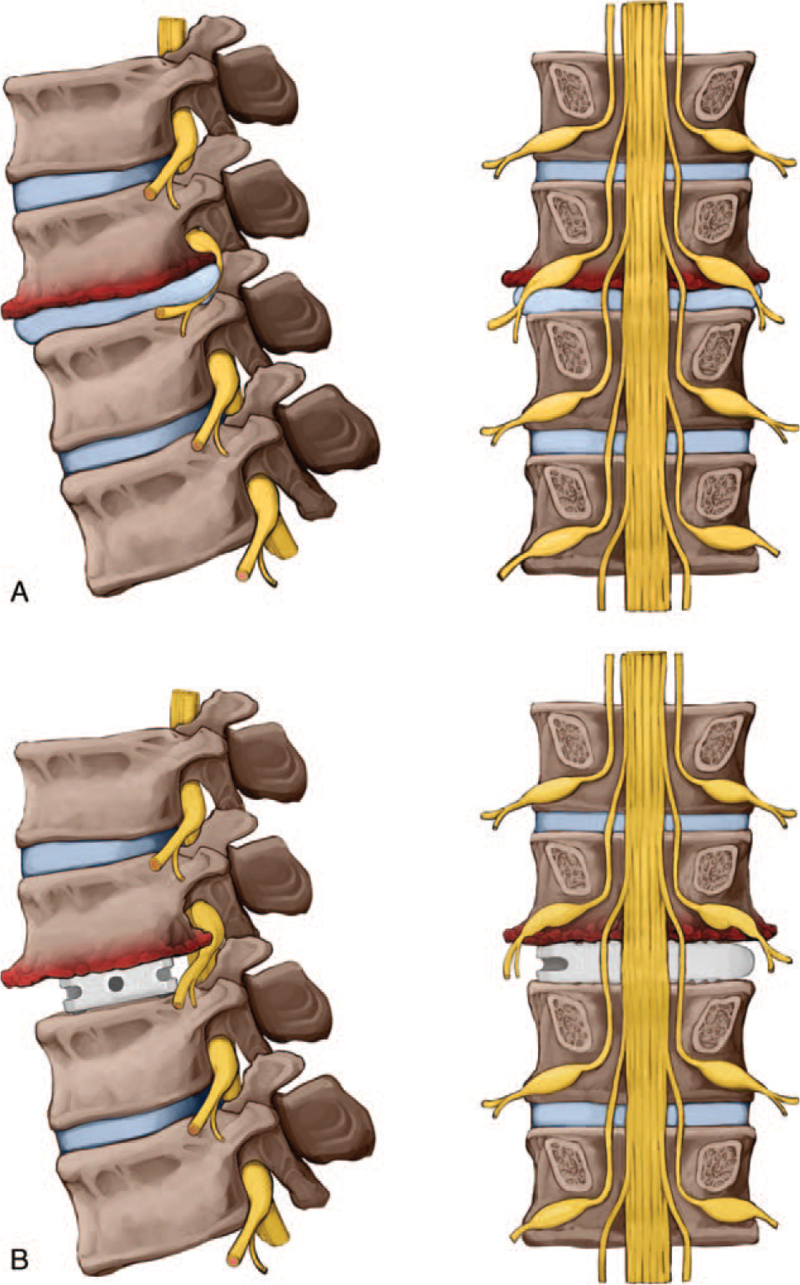
Illustration of our hypothesis that the nerve root is “intercalated” by the osteophytes after interbody fusion, resulting in injury. (A) Preoperative nerve root compression by disc herniation. (B) Disc height and foramen height increase after fusion. Consequently, the nerve root becomes elongated and is directed toward the body, and the osteophytes (red) of the upper lumbar vertebrae rise and trap the nerve roots.

Thus, we focused on the posterolateral protruding osteophytes of the inferior endplate of the L4 body in the coronal and sagittal planes. We hypothesized that the following mechanism are involved in the development of injury of the nerve root in relation to osteophytes after fusion. First, during indirect decompression, the nerves compressed by the disc are released, and the disc height and foraminal height increase with cage insertion. Second, this increase in height also causes elevation of the osteophytes. Third, owing to the elongation of the nerve root according to the disc height increase, the nerve root further moves toward the lumbar body to meet the osteophytes, and neurologic changes occur in the process of being “intercalated” (Fig. 3).

Silverstein et al^[20]^ suggests that the take-off angle of the nerve root is the greatest at L4 (range, 50.5°–57.2°) on the right side. When the disc height increases, the nerve root is stretched, and the take-off angle is reduced, the nerves with a large take-off angle are more vulnerable and can be easily intercalated in the osteophytes. Before OLIF, it is necessary to check for the presence and the shape of osteophytes on CT, and additional surgery should be considered depending on the shape observed. We also suggest that the more posteriorly located the osteophytes are, the longer they are, and that the sharper they are, the more likely they are to develop neurologic deficits due to these “intercalations.”

Our study has a limitation in that damage to the lumbar plexus or nerve root could not be confirmed during surgery because EMG or SSEP monitoring was not performed. In the future, it is necessary to investigate the possibility of postoperative nerve root intercalation according to the shape, length, and angle of the osteophyte, and the correlation between height change and fusion level in patients with osteophytes.

## 4 Conclusion

In our study, prominently protruding osteophytes were assessed as being a possible risk factor for the development of new neurologic deficits after OLIF. In patients with prominently protruding osteophytes, CT must be performed before and after surgery to distinguish the osteophytes from the discs to determine the relationship with the nerve roots. Further, additional laminectomy or foraminotomy after OLIF or other surgical methods, such as posterior lumbar interbody fusion, should be considered.

## Author contributions

**Conceptualization:** Bong Ju Moon.

**Data curation:** Tae-Kyu Lee, Jae-Young Kim.

**Formal analysis:** Jae-Young Kim, Moon-Soo Han.

**Investigation:** Tae-Kyu Lee, Jae-Young Kim, Moon-Soo Han, Bong Ju Moon.

**Supervision:** Jung-Kil Lee, Bong Ju Moon.

**Validation:** Jung-Kil Lee.

**Visualization:** Tae-Kyu Lee, Jung-Kil Lee.

**Writing – original draft:** Tae-Kyu Lee.

**Writing – review & editing:** Tae-Kyu Lee, Moon-Soo Han, Bong Ju Moon.
